# Three-dimensional simulation/printing-assisted surgery for symptomatic metastatic epidural spinal cord compression of posterior column: efficacy assessment based on 2-year follow-up

**DOI:** 10.3389/fsurg.2023.1177280

**Published:** 2023-05-26

**Authors:** Zhicheng Sun, Runze Jia, Xiyang Wang, Xiaoyang Pang

**Affiliations:** Department of Spinal Surgery, Xiangya Hospital of Central South University, Changsha, China

**Keywords:** spinal tumors, three-dimensional simulation, three-dimensional printing, metastatic epidural spinal cord compression, surgery

## Abstract

**Background:**

Surgical intervention is necessary for resolving the symptoms of the spinal cord and nerve compression caused by symptomatic metastatic epidural spinal cord compression. However, surgeons are constantly seeking ways to improve surgical efficiency and safety. This study aims to evaluate the efficacy of 3D simulation/printing-assisted surgery for symptomatic metastatic epidural spinal cord compression of the posterior column.

**Methods:**

We retrospectively analyzed the clinical data of patients who underwent surgical treatment for symptomatic metastatic epidural spinal cord compression of the posterior column in our hospital from January 2015 to January 2020. The simulated group underwent a 3D digital simulation of the lesion area using imaging data before surgery. Twelve patients in the simulated group also received 3D printing, while the direct surgery group did not receive any 3D simulation or printing. All patients were followed up for at least 2 years. We collected clinical data, including operation time, intraoperative blood loss, pedicle screw adjustment rate, intraoperative fluoroscopy times, the incidence of dural injury and cerebrospinal fluid leakage, VAS score, postoperative neurological function improvement, and tumor recurrence. Statistical analysis was performed using SPSS23.0, and *P* < 0.05 was considered statistically significant.

**Results:**

A total of 46 patients were included in this study, with 20 in the simulated group and 26 in the non-simulated group. The simulated group had better operation time, intraoperative blood loss, screw adjustment rate, fluoroscopy times, and incidence of dural injury/cerebrospinal fluid leakage compared to the non-simulated group. The VAS scores of the two groups improved significantly after the operation and at the last follow-up compared to before the operation. However, there was no statistically significant difference between the two groups. There was also no statistically significant difference in neurological function improvement between the two groups. In the simulated group, 25% of patients relapsed, while in the non-simulated group, 34.61% of patients relapsed. However, there was no statistical difference between the two groups.

**Conclusion:**

Preoperative 3D simulation/printing-assisted surgery is a practical and feasible approach for treating symptomatic metastatic epidural spinal cord compression of the posterior column.

## 1. Introduction

Spinal metastases are secondary tumors that have spread from primary cancer in another part of the body to the spine, with only about 1/5 involving the spinal appendage. Common primary tumor types include lung cancer, breast cancer, prostate cancer, liver cancer, kidney cancer, and cervical cancer (1). Treatment options for spinal metastases may include pain management, radiation therapy, surgery, and chemotherapy (2). Surgical intervention for spinal metastases is typically necessary when the tumors cause significant pain, pathological fracture, nerve compression, or other symptoms that affect the patient's quality of life (3).

The incidence of metastatic epidural spinal cord compression (MESCC) accounts for 5% to 10% of all malignancies (4). About one in ten patients with spinal metastases develops epidural compression disease (5), and approximately one-third of MESCC patients eventually experience neurological deficits that prevent them from walking (6). The standard treatment for MESCC consists of decompressive surgery and conventional radiotherapy (7). However, growing interest in minimally invasive techniques and stereotactic radiosurgery could revolutionize the development of spinal metastases treatment over the next decade (8). Nevertheless, since the tumor is located in proximity to the spinal cord and nerves, the surgery is highly susceptible to complications such as cerebrospinal fluid leakage, massive bleeding, and residual tumor, which can easily lead to nerve damage or paralysis. To reduce or avoid these complications during the perioperative period (9) and maximize the safety and effectiveness of surgical resection of spinal metastases, reliable auxiliary techniques such as three-dimensional (3D) simulation/printing are necessary options.

3D simulation/printing effectively uses CT imaging data to project human tissues and organs outside the body, creating visual three-dimensional digital models or physical models with equal proportions. These techniques display 3D information about the human body structure with higher resolution (10), providing more intuitive clinical information for clinicians. 3D printing, also known as additive manufacturing, is a deeper manifestation of 3D modeling. Charles Hull first described this process in 1986 (11) and involves joining materials to create objects from 3D model data (12). The unique 3D printing manufacturing technology allows for the production of items with unprecedented shapes and sizes, while maintaining geometric accuracy and complexity of the body (13). However, the practicality of 3D simulation/printing in surgery is still difficult to evaluate due to the lack of intuitive evaluation indicators. Therefore, this study aims to explore the value of 3D simulation/printing-assisted surgery in the treatment of metastatic epidural spinal cord compression of the posterior column from multiple outcome indicators.

## 2. Methods and materials

### 2.1. Objects

We conducted a retrospective analysis of clinical data from patients with metastatic epidural spinal cord compression of the posterior column who underwent surgical treatment at our hospital between January 2015 and January 2020. The inclusion criteria were as follows: (1) confirmation of spinal metastases by CT-guided biopsy; (2) symptoms of epidural spinal cord compression, including adicular pain, ataxia, motor weakness, sensory disturbances, and/or bladder dysfunction; (3) lesions affecting only the posterior column of the spine, not the anterior and middle columns; (4) Involvement of no more than 2 segments without multiple spinal metastases; (5) the patients were older than 18 years. (6) all surgical procedures were performed by the same medical staff; and (7) patients still alive at the last follow-up. A total of 46 patients were included in this study, with 20 cases in the simulated group and 26 cases in the non-simulated group. In the simulated group, all patients underwent a 3D digital simulation of the lesion area using imaging data before surgery. Of these, 12 patients received further 3D printing, while those in the non-simulated group did not undergo any simulation or printing. All patients were followed up for at least 2 years. This retrospective study was supported by the ethics committee of our institution.

### 2.2. Preoperative 3D simulation/printing

To obtain three-dimensional data of the spine, spine three-dimensional reconstruction computed tomography was used, and the data was imported into reconstruction software (Mimics Innovation Suite 21.0) to construct a 3D model. Different colors were used to identify the location and extent of the intraspinal tumors. This model was used to clarify the adjacent and surrounding invasion of the intraspinal tumors. Although 3D printing based on 3D modeling was optional for the patient because it was an additional expense, FARSOON-SS402P (Hunan Farsoon High-Technology Co., Ltd) was used for those who chose to undergo the procedure.

### 2.3. Operation procedure

The patients were placed in a prone position under general endotracheal anesthesia. A posterior median incision was made at the lesion site to expose the ackes, lamina, facet joints, and transverse processes. The metastasis location and boundary were then exposed and defined. With the help of C-arm fluoroscopy, the pedicle screw was inserted into the diseased segment vertebra, and the connecting rod was fixed to retain sufficient space for tumor resection. Laminectomy and adnexectomy were performed to remove the tumor with margin negative resection. We carefully separated the contact interface between the tumor and the dura and performed detailed blunt separation where the dura was surrounded and adhered. Bone grafting was then performed, followed by complete hemostasis and placement of a drainage tube. Finally, the surgical incision was closed, and specimens were collected for pathological examination.

### 2.4. Keynotes

Physicians who planned to participate in the operation carefully discussed and evaluated the 3D virtual model or 3D printed solid model one day before the operation. The evaluation included presetting reasonable screw entry points and screw entry directions, determining the negative resection margin of the tumor, identifying the tumor's adjacent relationship with important blood vessels and nerves, and predicting the risk of tumor resection and decompression based on the degree of tumor compression on the dura. Half an hour before the start of the operation, all physicians participating in the operation would re-evaluate the operation plan based on the 3D virtual model or 3D printed solid model. During the operation, it was particularly important to expose the key anatomical structures of the patient's surgical site. During the pedicle screw insertion, the surgeon compared the actual screw insertion point and direction with the 3D model. If there was an obvious deviation, it needed to be re-evaluated. During tumor resection, the surgeon first observed the tumor boundary with the naked eye, then compared it with the preset tumor-negative resection boundary in the 3D model, and made reasonable corrections to the resection boundary. During dura mater decompression, the more oppressed the dura mater was and the riskier the operation, the more careful the operation should be. Additionally, special attention should be given to the adhesion between the dura and the tumor.

### 2.5. Data collection and processing

Clinical data, including baseline information such as gender, age, lesion site, lesion levels, pathological sources, Spine Instability Neoplastic Score(SINS) (14) and follow-up time), operation time, intraoperative blood loss, intraoperative pedicle screw adjustment rate, intraoperative fluoroscopy times, the incidence of dural injury and cerebrospinal fluid leakage, VAS score, postoperative neurological function improvement, and tumor recurrence were collected from the two patient groups. SPSS23.0 was used to perform statistical analysis, and a *P* < 0.05 was considered statistically significant. Continuous data with a normal distribution were analyzed using a t-test, categorical data were analyzed using the chi-square test, continuity-corrected chi-square test, or Fisher's exact probability method, and graded data were analyzed using the Mann–Whitney *U*-test.

## 3. Results

### 3.1. Baseline data

**Figure 1:**
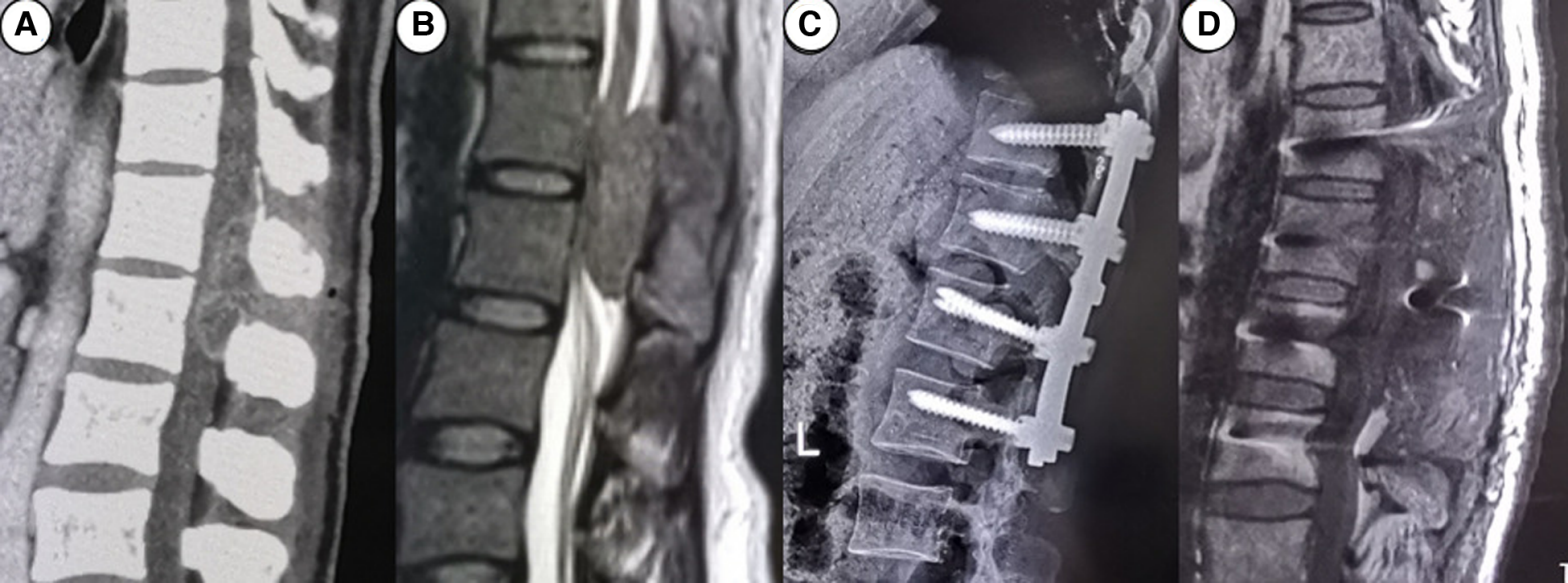
A 51-year-old woman with lung cancer metastases to the T11,12 posterior column, combined with epidural spinal cord compression. (**A**) the preoperative CT image; (**B**) the preoperative MRI image; (**C**) the postoperative CT image; (**D**) the postoperative MRI image.

**Figure 2:**
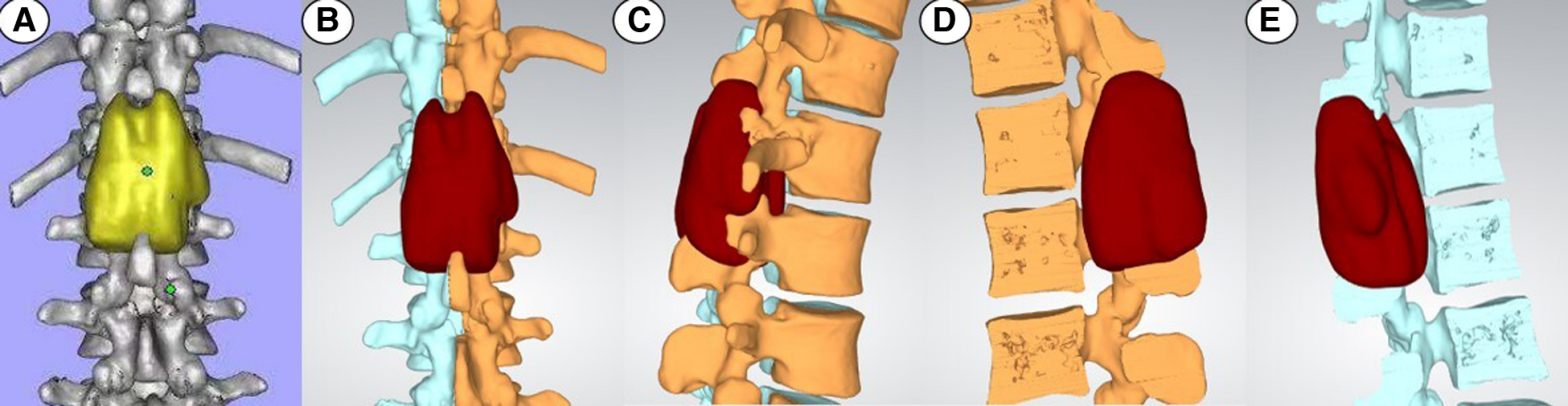
Figure 2A–2E showed the 3D simulation of lesion segments at different viewing angles.

Table 1 summarizes the demographics and disease characteristics of the patients. There were no significant differences in age, gender, tumor levels, tumor sources, SINS, and follow-up time between the two groups. All tumors were located in the thoracic spine, with pathological sources including lung cancer, prostate cancer, breast cancer, and other cancer types (such as liver cancer and thyroid cancer). All patients received conventional radiotherapy after surgery. Figures 1, 2 show radiographic and three-dimensional simulated image data of a 51-year-old woman with lung cancer metastases to the T11,12 posterior column, combined with epidural spinal cord compression.

**Table 1:** Baseline data of simulated group and non-simulated group.

Baseline data	Simulated group (*n *= 20	Non-simulated group (*n* = 26)	*P* value
Age	55.45 ± 7.57	56.58 ± 6.44	0.589
**Gender**			
Male	12	14	0.676
Female	8	12	
**Tumor levels**			
1	14	17	0.741
2	6	9	
**Tumor sources**			
Lung cancer	9	12	0.899
Prostate cancer	4	3	
Breast cancer	3	5	
Others	4	6	
SINS	8.05 ± 0.83	8.12 ± 0.71	0.775
Follow-up time	29.80 ± 4.93	30.62 ± 4.87	0.578

### 3.2. Clinical outcomes

The simulated group had an operation time of 1.94 + 0.29 h, and intraoperative blood loss of 382.50 + 94.97 ml. In contrast, the non-simulated group had an operation time of 2.15 + 0.25 h and intraoperative blood loss of 478.85 + 125.83 ml. In the simulated group, a total of 146 pedicle screws were implanted, with a screw adjustment rate of 12.33% and an average of 1.30 + 0.47 intraoperative fluoroscopy times. In contrast, the non-simulation group had a total of 186 pedicle screws implanted, with a screw adjustment rate of 21.50% and an average of 1.73 + 0.67 intraoperative fluoroscopy times. Three patients in the simulated group experienced dural damage/cerebrospinal fluid leakage, while 11 patients in the non-simulated group did. None of the patients experienced hematoma, internal fixation failure, internal fixation loosening or fracture, wound infection, bone graft nonunion, and lower extremity venous thrombosis. The statistical analysis indicated that the operation time, intraoperative blood loss, screw adjustment rate, radiographic times, and incidence of dural damage/cerebrospinal fluid leakage were all better in the simulated than in the non-simulated group. During the two-year follow-up period, 25% of patients in the simulated group and 34.61% in the non-simulated group experienced a relapse, but there was no statistical difference between the two groups. The results are shown in Table 2.

**Table 2:** Comparison of clinical outcomes between simulated group and non-simulated group.

Clinical outcomes	Simulated group (*n *= 20)	Non-simulated group (*n *= 26)	*P* value
Operation time (h)	1.94* *±* *0.29	2.15* *±* *0.25	0.01
Intraoperative blood loss (ml)	382.50* *±* *94.97	478.85* *±* *125.83	0.007
**Screw adjustment**			
Adjusted	18	40	0.025
Non-adjusted	128	146	
Intraoperative fluoroscopy times	1.30* *±* *0.47	1.73* *±* *0.67	0.018
**Dural damage/cerebrospinal fluid leakage**			
Yes	3	11	0.046
No	17	15	
Relapse			
Yes	5	9	0.482
No	15	17	

### 3.3. Pain and neurological assessment

Both groups presented with varying degrees of low back pain symptoms before the operation. Fortunately, after surgical treatment, these symptoms were significantly relieved compared to preoperative levels. However, there was no significant difference in VAS pain scores before the operation and 1 week after the operation between the two groups. The patients in both groups had varying degrees of spinal cord hypofunction due to dura compression before the operation, but there was no neurological deterioration after the operation. Neurological function was assessed using the ASIA grading method, and the postoperative neurological function improvement was graded as follows: significant improvement (ASIA grading increased by 2 levels), slight improvement (ASIA grading increased by 1 level), or no improvement (ASIA grading maintained at the same level). The results showed that there was no significant difference in the improvement of neurological function between the two groups. Table 3 shows the degree of improvement in VAS score and ASIA grading.

**Table 3:** Comparison of VAS score and ASIA grading between simulated group and non-simulated group.

	Simulated group (*n *=* *20)	Non-simulated group (*n *=* *26)	*P* value
**VAS score**			
Pre-operation	6.40* *±* *0.99	6.31* *±* *0.97	0.753
1 week after surgery	2.05* *±* *0.60*	2.15* *±* *0.67*	0.591
The last follow-up	1.90* *±* *0.64*△	1.88* *±* *0.52*△	0.928
**ASIA grading**			
Significant improvement	4	6	0.558
Small improvement	16	18	
No improvement	0	2	

* Indicates that the VAS score at 1 week after operation and at the last follow-up is significantly different from pre-operation.

△ means there is no significant difference between the VAS score at the last follow-up and the VAS score at 1 week after operation.

## 4. Discussion

The surgical methods for treating spinal metastases are rapidly developing, with minimally invasive techniques being a trend. Currently, minimally invasive techniques for spinal metastases treatment mainly include percutaneous instrumentation, mini-open approaches for decompression, and tumor removal with or without tubular/expandable retractors and thoracoscopy/endoscopy (15). Researchers believe that minimally invasive techniques can reduce blood loss, blood transfusion rates, and hospital stays compared to open surgery (16, 17). According to the latest research by Zhu Xiaojun et al., minimally invasive techniques for treating spinal metastases show less blood loss, less postoperative wound drainage, fewer postoperative complications and infections, and shorter hospital stay compared to traditional open surgery. This indicates that the minimally invasive technique is a safe and effective option for treating thoracolumbar metastases and can be considered an excellent option (18). Additionally, studies have shown that minimally invasive surgery for spinal tumors can lead to earlier postoperative radiotherapy. Radiotherapy and chemotherapy are often delayed after open surgery due to the risk of wounds (19). However, the quality of current literature data is not high, so the application is relatively limited and has not been widely promoted. Some scholars have made breakthrough attempts in navigation and robot-assisted spinal tumor resection (20). However, the promotion cost of this technology is high, the learning curve for surgeons is high, and it may also cause higher medical expenses for patients.

Although minimally invasive, navigation, and robotics technologies have significantly improved the efficiency and safety of spinal metastases resection, traditional open surgery remains an indispensable option for surgical treatment of MESCC, especially for giant tumors, multi-segment tumors, and tumors with complex anatomy. Palliative resection is a relatively traditional approach aimed at relieving nerve compression, rebuilding spinal stability, and improving the patient's quality of life. For solitary or short-segment vertebral metastases, the en-bloc technique has become a classic and mature approach, and its safety and efficacy have been recognized by the industry (21). However, perioperative complications of *en bloc* spinal resection should not be ignored, particularly in resection of more than two levels (22). In metastases involving only the appendages of the spine, a posterior laminectomy can also perform radical resection of the metastases without extensive en-bloc resection of the spine. In open surgery, several surgical interventions have emerged to improve the efficiency and safety of surgery, including percutaneous selective arterial embolization (23), multidisciplinary management (24), 3D printing technology (25), and intraoperative multimodal neurophysiological monitoring technology (26), and their feasibility has been preliminarily verified.

In the treatment of MESCC of the posterior column, the open posterior approach provides the surgeon with a clear view of the surgical site, enabling a comparison with the preoperative surgical planning based on 3D modeling/printing physical model. The use of 3D modeling/printing can provide additional information to surgeons, such as determining the tumor boundary, identifying the negative resection range, and assessing the relationship between the tumor and the dura, blood vessels, and nerves. However, when dealing with MESCC of the anterior and middle columns, the surgical field of view is often limited, restricting the surgeon's ability to follow a physical model-based surgical plan, even with the assistance of 3D modeling/printing. Therefore, this study emphasizes the usefulness of 3D simulation/printing technology in the treatment of MESCC of the posterior column.

Previous experience has shown that in the fields of neurosurgery and spine surgery, 3D printing can be used for anatomical training, surgical simulation, tumor biopsy, and resection, and for designing unique intraoperative implants and surgical equipment. In cases involving unfamiliar anatomical regions or complex neurovascular structures, virtual preoperative simulation can provide more valuable information than simple 2D images or limited CT-based 3D reconstructions (27). 3D printing-assisted surgery can reduce operation time, minimize intraoperative blood loss, and reduce the need for intraoperative radiological examinations (28). For complex spinal tumor cases, 3D printing can enhance preoperative planning, simplify surgical procedures, and improve reconstruction outcomes (29). The largest case series of 3D printed spinal tumor models reported by Leary OP et al. demonstrated the importance of 3D printing for surgical planning and real-time intraoperative guidance, based on the experience of a single center (30). Obtaining informed consent from the patient not only allows us to explain the condition but also disseminates the risks and facilitates intuitive communication (31, 32).

Our study focused on 3D simulation/printing-assisted surgery for symptomatic metastatic epidural spinal cord compression of the posterior column for the first time, and our results were consistent with previous studies. 3D simulation/printing can provide surgeons with effective preoperative planning, allowing for the prediction of key points and potential risks, thereby increasing surgical proficiency and accuracy during the operation. Our research showed that 3D simulation/printing assisted surgical treatment of symptomatic metastatic epidural spinal cord compression of the posterior column can reduce operation time, intraoperative blood loss, screw adjustment rate, fluoroscopy times, and incidence of dural injury/cerebrospinal fluid leakage, making the operation more efficient and safer. These results illustrate that 3D simulation/printing-assisted surgery is a practical and feasible option for treating symptomatic metastatic epidural spinal cord compression.

3D printing also has many disadvantages that cannot be ignored. For example, it is expensive and often not covered by national health insurance, making it less accessible for financially disadvantaged patients who may be reluctant to accept this additional expense. Furthermore, due to the limited availability of 3D printing technology in some hospitals, third-party cooperation may be necessary, leading to increased difficulty in communication between doctors and patients or potential patient mistrust. Consequently, the use of 3D printing is optional for patients at our institution. After receiving a sufficient explanation from the doctor, patients can weigh the pros and cons and make an informed choice. Therefore, the wider adoption of 3D printing technology still has a long way to go. Fortunately, 3D simulation can also meet clinical needs and achieve desirable outcomes.

Additionally, there is no clear evidence that preoperative 3D simulation/printing has a positive effect on tumor recurrence or survival time after resection. The decision of whether further radiotherapy/chemotherapy is necessary after surgery depends on the pathology of the metastatic tumor and the treatment status of the primary tumor. Several studies have demonstrated that preoperative adjuvant chemotherapy and postoperative further chemotherapy and radiotherapy may improve the prognosis and survival of patients with various types of spinal metastases (33–35).

## 5. Conclusion

The use of preoperative three-dimensional simulation/printing-assisted surgery is a practical and feasible approach for treating symptomatic metastatic epidural spinal cord compression. It can significantly reduce the operation time, intraoperative blood loss, screw adjustment rate, fluoroscopy times, and the incidence of dural injury and cerebrospinal fluid leakage.

## Data Availability

The raw data supporting the conclusions of this article will be made available by the authors, without undue reservation.
